# COVAD survey 2 long-term outcomes: unmet need and protocol

**DOI:** 10.1007/s00296-022-05157-6

**Published:** 2022-08-14

**Authors:** Zoha Zahid Fazal, Parikshit Sen, Mrudula Joshi, Naveen Ravichandran, James B. Lilleker, Vishwesh Agarwal, Sinan Kardes, Minchul Kim, Jessica Day, Ashima Makol, Marcin Milchert, Tamer Gheita, Babur Salim, Tsvetelina Velikova, Abraham Edgar Gracia-Ramos, Ioannis Parodis, Elena Nikiphorou, Ai Lyn Tan, Tulika Chatterjee, Lorenzo Cavagna, Miguel A. Saavedra, Samuel Katsuyuki Shinjo, Nelly Ziade, Albert Selva-O’Callaghan, Arvind Nune, Johannes Knitza, Masataka Kuwana, Carlos-Enrique Toro Gutiérrez, Carlo Vinicio Caballero-Uribe, Dzifa Dey, Oliver Distler, Hector Chinoy, Vikas Agarwal, Rohit Aggarwal, Latika Gupta, COVAD Study Group, Bhupen Barman, Yogesh Preet Singh, Rajiv Ranjan, Avinash Jain, Sapan C Pandya, Rakesh Kumar Pilania, Aman Sharma, Manesh Manoj M, Vikas Gupta, Chengappa G Kavadichanda, Pradeepta Sekhar Patro, Sajal Ajmani, Sanat Phatak, Rudra Prosad Goswami, Abhra Chandra Chowdhury, Ashish Jacob Mathew, Padnamabha Shenoy, Ajay Asranna, Keerthi Talari Bommakanti, Anuj Shukla, Arun Kumar R Pandey, Prithvi Sanjeevkumar Gaur, Mahabaleshwar Mamadapur, Akanksha Ghodke, Kunal Chandwar, Kshitij Jagtap, Zoha Zahid Fazal, Döndü Üsküdar Cansu, Reşit Yıldırım, Aarat Patel, John D Pauling, Chris Wincup, Margherita Giannini, François Maurier, Julien Campagne, Alain Meyer, Nicoletta Del Papa, Gianluca Sambataro, Atzeni Fabiola, Marcello Govoni, Simone Parisi, Elena Bartoloni Bocci, Gian Domenico Sebastiani, Enrico Fusaro, Marco Sebastiani, Luca Quartuccio, Franco Franceschini, Pier Paolo Sainaghi, Giovanni Orsolini, Rossella De Angelis, Maria Giovanna Danielli, Vincenzo Venerito, Silvia Grignaschi, Alessandro Giollo, Lisa S Traboco, Syahrul Sazliyana Shaharir, Suryo Anggoro Kusumo Wibowo, Erick Adrian Zamora Tehozol, Jorge Rojas Serrano, Ignacio García-De La Torre, Iris J. Colunga‑Pedraza, Iris J. Colunga‑Pedraza, Javier Merayo-Chalico, Jesús Loarce-Martos, Sergio Prieto-González, Albert Gil-Vila, Raquel Aranega, Leonardo Santos Hoff, Ran Nakashima, Shinji Sato, Naoki Kimura, Yuko Kaneko, Stylianos Tomaras, Fabian Nikolai Proft, Marie-Therese Holzer, Margarita Aleksandrovna Gromova, Or Aharonov, Melinda Nagy-Vincze, Zoltán Griger, Ihsane Hmamouchi, Pr Imane El bouchti, Zineb Baba, Uyi Ima-Edomwonyi, Ibukunoluwa Dedeke, Emorinken Airenakho, Nwankwo Henry Madu, Abubakar Yerima, Hakeem Olaosebikan, Okwara Celestine Chibuzo, Becky A, Ouma Devi Koussougbo, Elisa Palalane, Daman Langguth, Vidya Limaye, Merrilee Needham, Nilesh Srivastav, Marie Hudson, Océane Landon-Cardinal, Wilmer Gerardo Rojas Zuleta, Álvaro Arbeláez, Javier Cajas, José António Pereira Silva, João Eurico Fonseca, Olena Zimba, Doskaliuk Bohdana, Ho So, Manuel Francisco Ugarte-Gil, Lyn Chinchay, José Proaño Bernaola, Victorio Pimentel, A. T. M. Tanveer Hasan, Sreoshy Saha, Binit Vaidya, Hanan Mohamed Fathi, Reem Hamdy A Mohammed, Yi-Ming Chen, Ghita Harifi, Lina El Kibbi, Hussein Mohammed Halabi, P Akawatcharangura, Wanruchada Katchamart, Yurilís Fuentes-Silva, Karoll Cabriza, Jonathan Losanto, Nelly Colaman, Antonio Cachafeiro-Vilar, Generoso Guerra Bautista, Enrique Julio Giraldo Ho, Raúl Agustín González, Lilith Stange Nunez, Cristian Vergara M, Jossiell Then Báez, Hugo Alonzo, Carlos Benito Santiago Pastelin, Rodrigo García Salinas, Alejandro Quiñónez Obiols, Nilmo Chávez, Andrea Bran Ordóñez, Sandra Argueta, Daniel Quijivix, Gil Alberto Reyes Llerena, Radames Sierra-Zorita, Dina Arrieta, Eduardo Romero Hidalgo, Ricardo Saenz, Idania Escalante M., Roberto Morales, Wendy Calapaqui, Ivonne Quezada, Gabriela Arredondo

**Affiliations:** 1 Medical College, Aga Khan University Hospital, National Stadium Road, Karachi, 3500 Sindh Pakistan; 2 Maulana Azad Medical College, 2-Bahadurshah Zafar Marg, New Delhi, Delhi 110002 India; 3 Byramjee Jeejeebhoy Government Medical College and Sassoon General Hospitals, Pune, India; 4 Department of Clinical Immunology and Rheumatology, Sanjay Gandhi Postgraduate Institute of Medical Sciences, Lucknow, India; 5 Division of Musculoskeletal and Dermatological Sciences, School of Biological Sciences, Faculty of Biology, Medicine and Health, Centre for Musculoskeletal Research, Manchester Academic Health Science Centre, The University of Manchester, Manchester, UK; 6 Neurology, Manchester Centre for Clinical Neurosciences, Northern Care Alliance NHS Foundation Trust, Salford, UK; 7 Mahatma Gandhi Mission Medical College, Navi Mumbai, Maharashtra India; 8 Department of Medical Ecology and Hydroclimatology, Istanbul Faculty of Medicine, Istanbul University, Capa-Fatih, 34093 Istanbul, Turkey; 9 Department of Internal Medicine, Center for Outcomes Research, University of Illinois College of Medicine Peoria, Peoria, IL USA; 10 Department of Rheumatology, Royal Melbourne Hospital, Parkville, VIC 3050 Australia; 11 Walter and Eliza Hall Institute of Medical Research, Parkville, VIC 3052 Australia; 12 Department of Medical Biology, University of Melbourne, Parkville, VIC 3052 Australia; 13 Division of Rheumatology, Mayo Clinic, Rochester, MN USA; 14 Department of Internal Medicine, Rheumatology, Diabetology, Geriatrics and Clinical Immunology, Pomeranian Medical University in Szczecin, ul Unii Lubelskiej 1, 71-252 Szczecin, Poland; 15 Rheumatology Department, Kasr Al Ainy School of Medicine, Cairo University, Cairo, Egypt; 16 Rheumatology Department, Fauji Foundation Hospital, Rawalpindi, Pakistan; 17 Department of Clinical Immunology, Medical Faculty, University Hospital “Lozenetz”, Sofia University St. Kliment Ohridski, 1 Kozyak Str., 1407 Sofia, Bulgaria; 18 Department of Internal Medicine, General Hospital, National Medical Center, La Raza”, Instituto Mexicano del Seguro Social, Av. Jacaranda S/N, Col. La Raza, Del. Azcapotzalco, C.P. 02990 Mexico City, Mexico; 19 Division of Rheumatology, Department of Medicine Solna, Karolinska Institutet and Karolinska University Hospital, Stockholm, Sweden; 20 Department of Rheumatology, Faculty of Medicine and Health, Örebro University, Örebro, Sweden; 21 Centre for Rheumatic Diseases, King’s College London, London, UK; 22 Rheumatology Department, King’s College Hospital, London, UK; 23 NIHR Leeds Biomedical Research Centre, Leeds Teaching Hospitals Trust, Leeds, UK; 24 Leeds Institute of Rheumatic and Musculoskeletal Medicine, University of Leeds, Leeds, UK; 25 Department of Rheumatology, Fondazione I.R.C.C.S. Policlinico San Matteo, Pavia, Italy; 26 Rheumatology Unit, Dipartimento di Medicine Interna e Terapia Medica, Università degli studi di Pavia, Pavia, Lombardy Italy; 27 Departamento de Reumatología Hospital de Especialidades Dr. Antonio Fraga Mouret, Centro Médico Nacional La Raza, IMSS, Mexico City, Mexico; 28 Division of Rheumatology, Faculdade de Medicina FMUSP, Universidade de Sao Paulo, Sao Paulo, SP Brazil; 29 Rheumatology Department, Saint-Joseph University, Beirut, Lebanon; 30 Rheumatology Department, Hotel-Dieu de France Hospital, Beirut, Lebanon; 31 Southport and Ormskirk Hospital NHS Trust, Southport, PR8 6PN UK; 32 Systemic Autoimmune Diseases Unit, Medicine Department, Vall d’Hebron General Hospital, Universitat Autónoma de Barcelona, Barcelona, Spain; 33 Medizinische Klinik 3 - Rheumatologie und Immunologie, Universitätsklinikum Erlangen, Friedrich-Alexander-Universität Erlangen-Nürnberg, Ulmenweg 18, 91054 Erlangen, Germany; 34 Department of Allergy and Rheumatology, Nippon Medical School Graduate School of Medicine, 1-1-5 Sendagi, Bunkyo-ku, Tokyo, 113-8602 Japan; 35 Internal Medicine and Rheumatology, Reference Center for Osteoporosis, Rheumatology and Dermatology, Pontifica Javeriana University, ubicado en la Cra 77A# 5B 5-96 B, Cali, Colombia; 36 Department of Medicine, Hospital Universidad del Norte, Barranquilla, Colombia; 37 Department of Medicine and Therapeutics, College of Health Sciences, University of Ghana School of Medicine and Dentistry, Korle-Bu, Accra, Ghana; 38 Department of Rheumatology, University Hospital Zurich, University of Zurich, Zurich, Switzerland; 39 Division of Musculoskeletal and Dermatological Sciences, School of Biological Sciences, Centre for Musculoskeletal Research, The University of Manchester, Manchester, UK; 40 National Institute for Health Research Manchester Biomedical Research Centre, Manchester University NHS Foundation Trust, The University of Manchester, Manchester, UK; 41 Department of Rheumatology, Salford Royal Hospital, Northern Care Alliance NHS Foundation Trust, Salford, UK; 42 Division of Rheumatology and Clinical Immunology, University of Pittsburgh School of Medicine, Pittsburgh, PA USA; 43 Department of Rheumatology, Royal Wolverhampton Hospital NHS Trust, Wolverhampton, WV10 0QP UK; 44 City Hospital, Sandwell and West Birmingham Hospitals NHS Trust, Birmingham, UK

**Keywords:** COVID-19, Registries, Vaccination, Long-term adverse effects, Autoimmune diseases

## Abstract

Vaccine hesitancy is considered a major barrier to achieving herd immunity against COVID-19. While multiple alternative and synergistic approaches including heterologous vaccination, booster doses, and antiviral drugs have been developed, equitable vaccine uptake remains the foremost strategy to manage pandemic. Although none of the currently approved vaccines are live-attenuated, several reports of disease flares, waning protection, and acute-onset syndromes have emerged as short-term adverse events after vaccination. Hence, scientific literature falls short when discussing potential long-term effects in vulnerable cohorts. The COVAD-2 survey follows on from the baseline COVAD-1 survey with the aim to collect patient-reported data on the long-term safety and tolerability of COVID-19 vaccines in immune modulation. The e-survey has been extensively pilot-tested and validated with translations into multiple languages. Anticipated results will help improve vaccination efforts and reduce the imminent risks of COVID-19 infection, especially in understudied vulnerable groups.

**Supplementary Information:**

The online version contains supplementary material available at 10.1007/s00296-022-05157-6.

## The future of the COVID-19 pandemic: emerging problems and the way forward

It is unclear when, if and how natural regression of the ongoing coronavirus disease 2019 (COVID-19) pandemic, which has been a cause of unprecedented global morbidity and mortality, will be achieved. Moreover, the progression of COVID-19 to a chronic endemic illness is likely to increase the burden of emerging problems such as post-COVID-19 syndrome [1]. Preventive measures such as vaccination, can help reduce the socioeconomic and health burden associated with COVID-19 [2]. Thus, stringent advocacy for equitable vaccine uptake could help relieve the strain on over-stretched healthcare delivery systems, some of which have been pushed to the brink of collapse [3]. Vaccine efficacy, the longevity of immunity, the potency of booster doses, and the emergence of new severe acute respiratory syndrome coronavirus 2 (SARS-CoV-2) variants are considered important determinants in achieving herd immunity against COVID-19 [4].

Scientific ingenuity combined with global collaboration has accelerated the development, licensure, and dissemination of SARS-CoV-2 vaccines at an unprecedented pace. The milestone of 10 billion inoculations worldwide was already achieved in January 2022 [5] although there still remains considerable heterogeneity in the availability and uptake of vaccines across different regions. Nearly ten vaccines have been granted emergency use authorization (EUA), in addition to many vaccine candidates undergoing clinical trials and even more in the pre-clinical phase [6, 7]. Hence, appraising the protective capacity, dynamics, and persistence of immune responses following SARS-CoV-2 infection against different variants warrants rigorous research, especially with scarce evidence-base available.

## Navigating uncharted waters: COVID-19 vaccination in patients with autoimmune diseases

Several health organizations have recently authorized booster vaccine doses for previously vaccinated patients [8]. However, the differing administration criteria and scarcity of data on the safety and efficacy profile of these booster doses have led to speculations about booster safety and effectiveness in population groups with altered immunogenic responses. This specifically applies to the immunocompromised, those with autoimmune diseases, and patients undergoing immunotherapy. Heterologous vaccination is also a novel strategy to induce stronger immunogenic responses with acceptable reactogenicity profiles in the face of global vaccine supply interruptions and transmission of emerging variants [9]. Interestingly, this ‘mix and match’ vaccination approach has yielded 20- to 60-fold greater titers of neutralizing antibodies against the SARS-CoV-2 variants by including diverse spike sequences [10].

Recent data from the ongoing COVID-19 vaccination in autoimmune disease (COVAD) study [11] and other large alliance-based research projects have shown the short-term adverse effects of COVID-19 vaccination in patients with autoimmune diseases are comparable to those in healthy controls. Yet, vaccine hesitancy continues to be an impediment to achieving mass immunisation, including vulnerable patient groups [12]. Vaccine hesitancy is mainly contingent on expedited vaccine development and authorization, social media misinformation, distrust in health authorities, perceived adjunctive risks, certain religious beliefs, conspiracy theories, limited research, and assessment, mobilised opposition, and strategic politicisation against mass immunisation efforts [13]. Whilst certain nations, e.g. Brazil, South Africa, Denmark, and the UK are highly receptive to vaccine uptake with approximately 80% vaccination rates, they constitute only 10–20% of the global population [14]. The World Health Organization has rightly identified vaccine hesitancy as one of the top ten global health threats, with discrepancies in confidence, complacency, and convenience as key background players [15]. Vaccine hesitancy is especially a concern in understudied and vulnerable populations, such as pregnant and lactating women, who were largely excluded from the initial vaccine safety trials. While additional data are required to determine the long-term effects of maternal reactogenicity, current evidence strongly suggests that the benefits of maternal vaccination outweigh the theoretical risks [16].

While newer antiviral agents, such as Molnupiravir, Paxlovid, and Sotrivimab, are currently in use, their efficacy in patients with specific autoimmune diseases is yet to be ascertained [17]. Despite the favourable adverse drug event (ADE) profile of SARS-CoV-2 vaccines, there have been concerns regarding the activation of aberrant immune responses. The adjuvants of inoculation can induce hypersensitivity reactions, and those with a heightened immune response or with an immunocompromised status may respond differently [18]. Since none of the currently authorised COVID-19 vaccines are live, the Centers for Disease Control and Prevention (CDC) considers them safe for administration to individuals with modulated immunity although with variable levels of protection [19]. However, skepticism regarding the long-term safety and efficacy of the vaccines, with reports of waning protection over a few months, the emergence of new autoimmune diseases as per several reported cases [20], and the exclusion of susceptible populations from phased vaccine administration have instilled concerns about vaccine safety and tolerability in this group [12]. Preliminary data from recent large-scale alliance-based studies suggest that the short-term effects of vaccination are comparable in patients with autoimmune disease and healthy controls and that the benefits of vaccination in reducing the severity of COVID-19 infection outweigh the risk of post-vaccination adverse effects in cases of immunomodulation [21].

## A step towards meaningful data: the COVID-19 vaccination in autoimmune diseases survey

The paucity of evidence-based literature on the short-term safety of COVID-19 vaccines in patients with autoimmune diseases aroused distrust amongst these individuals despite the widely-asserted safety of vaccines [12]. To address this, the COVAD study was conceived, as a patient-reported international electronic survey, to collect meaningful data on the safety and tolerability of COVID-19 vaccines in patients with autoimmune diseases and healthy individuals. The baseline survey featured 36 questions, was hosted on the surveymonkey.com platform, translated into 18 languages, and circulated in early 2021 by the global COVAD study group of 110 physicians in over 94 countries. The initial survey collected over 19,200 responses that helped compile meaningful data on the short-term safety profile of COVID-19 vaccination [11]. However, questions regarding the long-term safety of COVID-19 vaccination remained unanswered.

## Unanswered questions: the 2nd COVAD survey

Thereafter, the COVAD-2 survey was formulated with a similar methodology for its development, dissemination, and data collection as the baseline COVAD-1 survey to build on the information previously collected while preserving the original core item set. COVAD-2 will thus serve as a follow-up survey for past respondents and a standalone survey for new participants. The COVAD-2 e-survey has been vetted and extensively pilot-tested by a group of international experts including rheumatologists, neurologists, and internists. It has further undergone testing in patients and lay public and medical students to ensure the questions are simple and easy to understand. It is being translated into several languages and will then be hosted on the online platform surveymonkey.com followed by dissemination by the international expert COVAD study team on social media platforms (e.g. WhatsApp, Facebook, Instagram, and Twitter) and among patient groups in the out patient clinics. Special efforts are being made to cover regions of the world that were relatively under-represented in the current literature and COVAD-1 survey, including Africa, South America, and several countries in Asia.

This ongoing patient-reported study follows the predefined guidelines for survey-based reporting and is conceptualised to gather global data on the long-term efficacy of vaccines [22, 23]. Moreover, it will answer additional questions not previously explored in the baseline survey, such as the potential of vaccine-induced disease flares, de novo emergence of autoimmune diseases, effects of booster vaccine doses, and specific risks of antenatal vaccination. A more in-depth insight into the effects of vaccination on the functional status and quality of life of patients with autoimmune diseases, particularly idiopathic inflammatory myopathies, as well as pregnant and lactating women will also be acquired. Using the Patient-Reported Outcomes Measurement Information System (PROMIS) global and physical function 10A will offer the opportunity to objectively quantify the impact of breakthrough infections and even long COVID-19 [24]. The COVAD-2 survey questionnaire is included in Supplementary File 2.

Data will be downloaded from surveymonkey.com in Microsoft Excel format and imported into statistical analysis packages for further analysis. Descriptive statistics will be used with intergroup comparisons ad hoc using Stata. Open-ended responses will be qualitatively analysed with manual allocation to pre-existent or new categories while incomplete responses will be excluded. The anonymized dataset will be open to future analysis of proposed hypotheses, research questions, and study designs approved by collaborators of the COVAD steering committee for scientific validity and feasibility.

Results will be disseminated in select peer-reviewed journals, on virtual platforms, and at academic conferences. A summary of the study outcomes in plain language will be provided to the respondents upon request. All information about the project, such as study design, project administration, and publication preprints, will be made available upon request. At the conclusion of the study, recruitment materials, project destination links, and online survey media will be deactivated or removed. All data will remain confidential with the principal investigator for a period of five years.

## Bridging the gap: bringing patients and healthcare providers closer in the era of COVID-19

Although the switch to remote consultations during the COVID-19 pandemic has widened the communication gap between healthcare providers and patients, it is imperative to address patient concerns and adverse events following vaccination as gathered by this self-reported survey. The results will help improve vaccination efforts and provide future policymakers with valuable data to formulate immunisation guidelines and reduce the imminent risks of COVID-19 in high-risk immunosuppressed groups that are currently understudied. Collaborative efforts toward rebuilding public confidence and community engagement with research-backed communication are essential for improving vaccine acceptance among those now holding views of vaccine hesitancy. The COVAD-2 survey thus follows a similar methodology for its development, dissemination, and data collection as the baseline COVAD-1 survey and aims to promote evidence-based health literacy in immunologically vulnerable populations.

## Supplementary Information

Below is the link to the electronic supplementary material.

Supplementary file1 (DOCX 51 KB)

Supplementary file2 (PDF 897 KB)
