# Supplementary material for: COVAD survey 2 long-term outcomes: unmet need and protocol
Source: Rheumatol Int. 2022 Aug 14;42(12):2151–8. doi: 10.1007/s00296-022-05157-6 (PMC9376047; doi:10.1007/s00296-022-05157-6)
Supplement: Supplementary file 1 — Supplementary file1 (DOCX 51 KB) [file 296_2022_5157_MOESM1_ESM.docx]

Supplementary File 1. COVID-19 Vaccination in Autoimmune Diseases-2 (COVAD-2) Study Group Author List and Affiliations

**India**

1. **Dr Bhupen Barman**: Department of  Medicine, North Eastern Indira Gandhi Regional Institute of Health and Medical Sciences(NEIGRIHMS), Shillong-18, Meghalaya, India
2. **Dr Yogesh Preet Singh**: Dr. Yogesh Preet Singh, Assistant Professor, Division of Rheumatology and Clinical Immunology, Department of General Medicine, Himalayan Institute of Medical sciences, Swami Rama University, Jolly Grant, Dehradun - 248140, Uttarakhand, India
3. **Dr Rajiv Ranjan**: Clinical Immunology & Rheumatology at Columbia Asia, Palam Vihar, Gurgaon, Haryana, India
4. **Dr Avinash Jain**: Department of Clinical Immunology and Rheumatology, SMS Medical College and Hospital, Jaipur, Rajasthan, India
5. **Dr Sapan C Pandya**: Rheumatic Disease Clinic, Vedanta Institute of Medical Sciences, Navrangpura, Ahmedabad 380009, Gujarat, India
6. **Dr Rakesh Kumar Pilania**: Pediatric Allergy Immunology Unit, Department of Pediatrics, Post Graduate Institute of Medical Education and Research, Chandigarh, India
7. **Dr Aman Sharma**: Clinical Immunology and Rheumatology Services, Department of Internal Medicine, Post Graduate Institute of Medical Education and Research, Chandigarh, India
8. **Dr Manesh Manoj M**: Department of Clinical Immunology and Rheumatology, AKG Memorial Hospital and Dr Shenoy’s CARE (Centre for Arthritis and Rheumatism Excellence), Kannur, Kerala, India
9. **Dr Vikas Gupta**: Rheumatology, Dayanand Medical College and Hospital, Ludhiana, Punjab 141001, India
10. **Dr Chengappa G Kavadichanda**: Department of Clinical Immunology, Jawaharlal Institute of Postgraduate Medical Education and Research, Puducherry, India
11. **Dr Pradeepta Sekhar Patro**: Department of Clinical Immunology and Rheumatology, Sunshine Hospitals, Plot No. 208, Cuttack Puri Road, Laxmisagar, Bhubaneshwar, Odisha, India
12. **Dr Sajal Ajmani**: Arthritis and Rheumatology clinic, New Delhi, Delhi, India
13. **Dr Sanat Phatak**: Rheumatology & Immunology, Department of Rheumatology and Immunology, KEM Hospital, Pune, Maharashtra, India
14. **Dr Rudra Prosad Goswami**: Department of Rheumatology, All India Institute of Medical Sciences, New Delhi, Delhi, India
15. **Dr Abhra Chandra Chowdhury**: Rheumatology, AMRI Hospital, Dhakuria, Kolkata, West Bengal, India
16. **Dr Ashish Jacob Mathew**: Department of Clinical Immunology & Rheumatology, Christian Medical College and Hospital, Vellore, Tamil Nadu 632004, India
17. **Dr Padnamabha Shenoy**: Dr Shenoy’s CARE (Centre for Arthritis and Rheumatism Excellence), Kannur, Kerala, India
18. **Dr Ajay Asranna**: Department of Neurology, NIMHANS, Bengaluru, Karnataka, India
19. **Dr Keerthi Talari Bommakanti**: Rheumatology, Yashoda hospital, Behind Hari Hara Kala Bhavan, Secunderabad - 500003, T.S. Hyderabad, Telangana, India
20. **Dr Anuj Shukla**: Niruj Rheumatology Clinic, 209 Rajvi Complex, Rambaug, Ahmedabad, 380008, Gujarat, India
21. **Dr Arun Kumar R Pandey:** LEDTC Clinic, Gomti Nagar, Lucknow, Uttar Pradesh, India
22. **Prithvi Sanjeevkumar Gaur:** Smt. Kashibai Navale Medical and General Hospital, Pune, India
23. **Dr Mahabaleshwar Mamadapur:** Department of Clinical Immunology and Rheumatology, Sanjay Gandhi Postgraduate Institute of Medical Sciences, Lucknow, India
24. **Akanksha Ghodke**: Mahatma Gandhi Mission Medical College, Navi Mumbai, Maharashtra, India
25. **Dr Kunal Chandwar**: Department of Clinical Immunology and Rheumatology, King George's Medical University, Lucknow, Uttar Pradesh, India
26. **Kshitij Jagtap**: Seth Gordhandas Sunderdas Medical College and King Edward Memorial Hospital, Mumbai, Maharastra, India

**Pakistan**

- - - 1. **Zoha Zahid Fazal:** Medical College, The Aga Khan University, Karachi, Pakistan

**Turkey**

1. **Dr Döndü Üsküdar Cansu**: Division of Rheumatology, Department of Internal Medicine, Eskişehir Osmangazi University, 26480, Eskişehir, Turkey
2. **Dr Reşit Yıldırım**: Division of Rheumatology, Osmangazi University, Turkey

**United States of America**

- - - 1. **Dr Aarat Patel**: Bon Secours Rheumatology Center and Division of Pediatric Rheumatology, Department of Pediatrics, University of Virginia School of Medicine , Charlottesville, VA, USA

**United Kingdom**

1. **Dr John D Pauling**: 1. Royal National Hospital for Rheumatic Diseases (at Royal United Hospitals), Upper Borough Walls, Bath, BA1 1RL, UK. 2. Department of Pharmacy and Pharmacology, University of Bath, Bath, UK
2. **Dr Chris Wincup**: Department of Rheumatology, Division of Medicine, Rayne Institute, University College London, 5 University Street, London WC1E 6JF, UK; Centre for Adolescent Rheumatology Versus Arthritis at UCL, UCLH, GOSH, London, UK

**France**

- - - 1. **Dr. Margherita Giannini**: Explorations fonctionnelles musculaires, service de physiologie, Hôpitaux universitaires de Strasbourg; EA3072, fédération de médecine translationnelle, France
      2. **Dr François Maurier:** Service de Médecine Interne, Hôpital Robert Schuman, Rue du Champ Montoy, 57070 Vantoux, France.
      3. **Dr Julien Campagne:** Service de Médecine Interne, Hôpital Robert Schuman, Rue du Champ Montoy, 57070 Vantoux, France.

1. **Dr Alain Meyer:** 1. Centre National de Référence des Maladies Systémiques et Auto-immunes Rares Grand-Est Sud-Ouest (RESO), Service de humatologie, Service de physiologie, Unité d’explorations fonctionnelles musculaires, Hôpitaux Universitaires de Strasbourg, Strasbourg, France; 2. EA3072, Fédération de Médecine Translationelle, Université de Strasbourg, Strasbourg, France

**Italy**

1. **Dr. Nicoletta Del Papa**: Unità operativa complessa (UOC) Day Hospital Reumatologia via Gaetano Pini 9, Centro Specialistico Ortopedico Traumatologico, Gaetano Pini-CTO, Milano, Italy
2. **Dr. Gianluca Sambataro**: Medico Immunologia e reumatologia presso, Artoreuma S.R.L., Cors S. Vito 53, 95030 Mascalucia, CT, Italy
3. **Dr. Atzeni Fabiola**: Rheumatology Unit,  University of Messina, Messina, Italy
4. **Dr. Marcello Govoni**: Department of Medical Sciences, Complex Operative Unit and Rheumatology Unit of S.Anna University Hospital, University of Ferrara, Via A. Moro 8, 44124- Cona (FE), Italy
5. **Dr Simone Parisi**: Epidemiology Unit, Italian Society for Rheumatology, Milan, Italy; Rheumatology Unit, Azienda Ospedaliera Città della Salute e della Scienza di Torino, Torino, Italy
6. **Dr Elena Bartoloni Bocci**: Department of Medicine and Surgery, MED/16- Rheumatology, Università degli studi di Perugia, P.zza Università - 06123 – Perugia, Italy
7. **Dr. Gian Domenico Sebastiani**: U.O.C. Reumatologia, Ospedale San Camillo-Forlanini, Roma, Italy
8. **Dr Enrico Fusaro**: Rheumatology Unit, Azienda Ospedaliero-Universitaria Città della Salute e della Scienza di Torino, Torino, Italy
9. **Dr Marco Sebastiani**: Rheumatology Unit, University of Modena and Reggio Emilia, Azienda Ospedaliero-Universitaria Policlinico di Modena, Via del Pozzo, 41125, Modena, Italy
10. **Dr Luca Quartuccio**: Clinic of Rheumatology, Department of Medicine (DAME), ASUFC, University of Udine, Udine, Italy
11. **Dr Franco Franceschini**: Rheumatology and Clinical Immunology Unit, Department of Clinical and Experimental Sciences, ASST Spedali Civili and University of Brescia, Italy
12. **Dr Pier Paolo Sainaghi**: 1. Department of Translational Medicine, Università del Piemonte Orientale UPO, Novara, Italy. 2. Division of Internal Medicine, Immunorheumatology Unit, CAAD (Center for Translational Research on Autoimmune and Allergic Disease) Maggiore della Carità Hospital, Novara, Italy. 3. IRCAD, Interdisciplinary Research Center of Autoimmune Diseases, Novara, Italy
13. **Dr Giovanni Orsolini**: Department of Medicine, Rheumatology Unit, University of Verona, Verona, Italy
14. **Dr Rossella De Angelis**: Rheumatology Unit, Department of Clinical and Molecular Sciences, Polytechnic University of Marche, Italy
15. **Dr Maria Giovanna Danielli**: Clinica Medica, Dipartimento di Scienze Cliniche e Molecolari, Università Politecnica delle Marche e Azienda Ospedali Riuniti, Ancona, Italy
16. **Dr Vincenzo Venerito**: Department of Emergency and Organ Transplantations-Rheumatology Unit, University of Bari "Aldo Moro", Bari, Italy
17. **Dr Silvia Grignaschi**: Rheumatology Unit, Dipartimento di Medicine Interna e Terapia Medica, Università degli studi di Pavia, Pavia, Lombardy, Italy
18. **Dr. Alessandro Giollo.** Division of Rheumatology, Department of Medicine, University of Padova Hospital Trust, Padova, Italy.

**Philippines**

1. **Dr Lisa S Traboco**: Philippine Rheumatology Association, St Luke’s Medical Center- Global City (Visiting), Philippines

**Malaysia**

- - - 1. **Dr. Syahrul Sazliyana Shaharir**- Rheumatology Unit, Department of Internal Medicine, Universiti Kebangsaan Malaysia Medical Centre (UKMMC), Jalan Yaacob Latiff, Kuala Lumpur, Malaysia.

**Indonesia**

1. **Dr Suryo Anggoro Kusumo Wibowo**: Rheumatology Division, Department of Internal Medicine, Fakultas Kedokteran, Universitas Indonesia, Indonesia

**Mexico**

1. **Dr Erick Adrian Zamora Tehozol**: Centro Médico Pensiones, Autoimmunity Division, Mérida, Yucatán, Mexico
2. **Dr Jorge Rojas Serrano**: Interstitial Lung Disease and Rheumatology Unit, Instituto Nacional de Enfermedades Respiratorias, Mexico City, Mexico
3. **Dr Ignacio García-De La Torre:** Departamento de Inmunología y Reumatología, Hospital General de Occidente and University of Guadalajara, Guadalajara, Jalisco, Mexico
4. **Dr. Iris J. Colunga‑Pedraza**: Rheumatology Service, Facultad de Medicina y Hospital Universitario “Dr. JoseE. Gonzalez”, Universidad Autonoma de Nuevo Leon, Av. Francisco I. Madero yGonzalitos S/N, Colonia Mitras Centro, 64460 Monterrey, Nuevo Leon, Mexico
5. **Dr. Iris J. Colunga‑Pedraza**: Rheumatology Service, Facultad de Medicina y Hospital Universitario “Dr. JoseE. Gonzalez”, Universidad Autonoma de Nuevo Leon, Av. Francisco I. Madero yGonzalitos S/N, Colonia Mitras Centro, 64460 Monterrey, Nuevo Leon, Mexico
6. **Dr.** **Javier Merayo-Chalico**: Department of Immunology and Rheumatology, Instituto Nacional de Ciencias Médicas y Nutrición “Salvador Zubirán”, Mexico City, Mexico

**Spain**

1. **Dr Jesús Loarce-Martos**: Rheumatology Department, Hospital Universitario Ramón y Cajal, Carretera de Colmenar Viejo, 9, 1 km, 28043, Madrid, Spain
2. **Dr Sergio Prieto-González**: Department of Internal Medicine, Hospital Clinic of Barcelona, University of Barcelona, Barcelona, Spain
3. **Dr Albert Gil-Vila:** Systemic Autoimmune Diseases Unit, Vall d'Hebron General Hospital, Medicine Department, Universitat Autónoma de Barcelona, Barcelona, Spain
4. **Dr Raquel Aranega:** Systemic Autoimmune Diseases Unit, Vall d'Hebron General Hospital, Medicine Department, Universitat Autónoma de Barcelona, Barcelona, Spain

**Brazil**

1. **Dr Leonardo Santos Hoff:** School of Medicine, Universidade Potiguar (UnP), Brazil.

**Japan**

1. **Dr Ran Nakashima**: Department of Rheumatology and Clinical Immunology, Graduate School of Medicine, Kyoto University, 54 Shogoin-Kawahara-cho, Sakyo-ku, Kyoto 606-8507, Japan
2. **Dr Shinji Sato**: Division of Rheumatology, Department of Internal Medicine, Tokai University School of Medicine, 143 Shimokasuya, Isehara, 259-1193, Japan
3. **Dr Naoki Kimura**: Department of Lifetime Clinical Immunology, Graduate School of Medical and Dental Sciences, Tokyo Medical and Dental University (TMDU), Tokyo, Japan
4. **Dr Yuko Kaneko**: Division of Rheumatology, Department of Internal Medicine, Keio University School of Medicine, Tokyo, Japan

**Germany**

1. **Dr Stylianos Tomaras**: Department of Rheumatology, Helios Clinic Vogelsang-Gommern, 39245 Gommern, Germany
2. **Dr Fabian Nikolai Proft**: Department of Gastroenterology, Infectiology and Rheumatology (including Nutrition Medicine), Charité - Universitätsmedizin Berlin, corporate member of Freie Universität Berlin and Humboldt- Universität zu Berlin, Berlin, Germany
3. **Dr Marie-Therese Holzer**: 1. Department of Pediatrics, Pediatric Rheumatology/Special Immunology, University Hospital Wuerzburg, Josef-Schneider-Str. 2, 97080, Wuerzburg, Germany. 2. Department of Internal Medicine III. (Nephrology and Rheumatology With Section Endocrinology), University Hospital Hamburg- Eppendorf, University Hospital Hamburg-Eppendorf, Martinistraße 52, 20246, Hamburg, Germany

**Russian Federation**

1. **Dr Margarita Aleksandrovna Gromova**: Pirogov Russian National Research Medical University (RNRMU), Moscow, Russian Federation

**Israel**

1. **Mr Or Aharonov**: Department of Gerontology, Faculty of Social Welfare and Health Science, University of Haifa, Haifa, Israel

**Hungary**

1. **Dr Melinda Nagy-Vincze**: 1. Division of Clinical Immunology, Faculty of Medicine, University of Debrecen, Móricz Zsigmond út 22, Debrecen, H-4032, Hungary. 2. Gyula Petrányi Doctoral School of Clinical Immunology and Allergology, University of Debrecen, Debrecen, Hungary
2. **Dr Zoltán Griger**: 1. Division of Clinical Immunology, Faculty of Medicine, University of Debrecen, Móricz Zsigmond út 22, Debrecen, H-4032, Hungary

**Morocco**

1. **Dr Ihsane Hmamouchi:** Laboratoire d'épidémiologie et de recherche clinique, La Faculté de Médecine et de Pharmacie de Rabat, Morocco
2. **Dr Pr Imane El bouchti**: Rheumatology Department, Mohammed VI University Hospital, Marrakech, Morocco
3. **Dr. Zineb Baba**: Department of Rheumatology, Mohammed VI University Hospital, Marrakech, Morocco

**Nigeria**

1. **Dr Uyi Ima-Edomwonyi:** Consultant, Department of Internal Medicine, Lagos University Teaching Hospital, Lagos, Nigeria
2. **Dr Ibukunoluwa Dedeke**: Department of Medicine, University College Hospital Ibadan, Ibadan, Nigeria
3. **Dr Emorinken Airenakho**: Consultant Rheumatologist, Irrua Specialist Teaching Hospital, KM 87 Benin Auchi Rd, 310115, Irrua, Nigeria
4. **Dr Nwankwo Henry Madu**: Department of Medicine, Nnamdi Azikiwe University, Awka, Nigeria
5. **Dr Abubakar Yerima**: Department of Medicine, University of Maiduguri Teaching Hospital, Maiduguri, Borno State, Nigeria
6. **Dr Hakeem Olaosebikan**: Rheumatology, Lagos State University Teaching Hospital/ Lagos State University College of Medicine,Ikeja, Lagos, Nigeria
7. **Dr Okwara Celestine Chibuzo**: Rheumatology, University of Nigeria Teaching Hospital, Enugu, Nigeria

**Ethiopia**

- - - 1. **Dr Becky A:** Rheumatology Unit, Internal Medicine Department, Addis Ababa University, Addis Ababa, Ethiopia

**Mauritius**

**Dr Ouma Devi Koussougbo**: Rheumatology, Victoria hospital, Mauritius

**Mozambique**

**Dr Elisa Palalane**: Rheumatology and Internal Medicine, Hospital Central de Maputo, Maputo, Mozambique

**Australia**

1. **Dr Daman Langguth**: Department of Immunology, Sullivan Nicolaides Pathology, Brisbane, Queensland, Australia
2. **Dr Vidya Limaye**: Rheumatology, Royal Adelaide Hospital and Discipline of Medicine, University of Adelaide, Australia
3. **Dr Merrilee Needham**: 1. Neurology Department, Fiona Stanley Hospital, Murdoch, Australia; 2. Institute for Immunology and Infectious Diseases, Murdoch University, Murdoch, Australia; 3. Perron Institute for Neurological and Translational Science, Nedlands, Australia; 4. University of Notre Dame, Fremantle, Australia
4. **Dr Nilesh Srivastav**: Alfred Health, The Alfred, Caulfield Hospital, Sandringham Hospital, Melbourne, Victoria, Australia

**Canada**

- - - 1. **Dr Marie Hudson:** Department of Medicine, McGill University, Montreal, Quebec; Division of Rheumatology, Jewish General Hospital, Montreal, Quebec, and Lady Davis Institute, Jewish General Hospital, Montreal, Quebec, Canada
      2. **Dr Océane Landon-Cardinal:** 1. Department of Medicine, University of Montreal, Montreal, Canada. 2. Department of Medicine, CHUM Research Centre, Montreal, Canada

**Colombia**

- - - 1. **Dr Wilmer Gerardo Rojas Zuleta**- Department of Rheumatology, Universidad de Antioquia, Cl. 67 #53 - 108, Medellín, Colombia.

1. **Dr. Álvaro Arbeláez: Médico especialista en Reumatología y Medicina Interna. Universidad Libre. Clínica Imbanaco. Clínica de Artritis Temprana. Cali, Valle, Colombia.**
2. **Dr. Javier Cajas:** Institute of Rheumatology Ferdinand Chalem, Bogota, Colombia.

**Portugal**

- - - 1. **Dr José António Pereira Silva**: Rheumatology Department, Centro Hospitalar e Universitário de Coimbra EPE, and Coimbra Institute of Clinical and Biomedical Research (iCBR), Faculty of Medicine, University of Coimbra, Portugal
      2. **Dr João Eurico Fonseca**: Hospital de Santa Maria, Centro Hospitalar Lisboa Norte Centro Académico de Medicina de Lisboa, Lisboa, Portugal; Instituto de Medicina Molecular, Faculdade de Medicina, Universidade de Lisboa, Lisboa, Portugal

**Ukraine**

**Dr Olena Zimba:** Department of Internal Medicine #2, Danylo Halytsky Lviv National Medical University, Lviv, Ukraine

**Dr Doskaliuk Bohdana**: Department of Pathophysiology, Ivano-Frankivsk National Medical University, Ivano-Frankivsk, Ukraine

**Hong Kong**

1. **Dr Ho So**: Department of Medicine & Therapeutics, Faculty of Medicine, The Chinese University of Hong Kong, Hong Kong

**Peru**

1. **Dr Manuel Francisco Ugarte-Gil**: Servicio de Reumatología, Hospital Nacional Guillermo Almenara Irigoyen, EsSalud, Lima, Peru. School of Medicine, Universidad Científica del Sur, Lima, Peru
2. **Dr. Lyn Chinchay**: Seguro Social de Salud del Peru (ESSALUD), Lima, Peru
3. **Dr. José Proaño Bernaola**: 1. Cayetano Heredia National Hospital, Lima, Peru. 2. Anglo-American Clinic. San Isidro, Lima, Peru. 3. Peruvian University Cayetano Heredia, Lima, Peru. 4. Rheumatologist, San Judas Tadeo Clinic - Research Center (ENDOMED), Lima. 5. Environmental and Food Health - DIRIS – Lima, Peru
4. **Dr. Victorio Pimentel**: Rheumatology Department, Hospital Guillermo Almenara Irigoyen, EsSalud, Av. Grau 800, La Victoria, Lima 13, Lima, Peru

**Bangladesh**

1. **Dr A.T.M. Tanveer Hasan**: Department of Rheumatology, Enam Medical College and Hospital, 9/3 Parboti Nagar, Thana Rd, Savar Union 1340, Bangladesh
2. **Sreoshy Saha**: Mymensingh Medical College, Mymensingh, Bangladesh

**Nepal**

1. **Dr Binit Vaidya**: Department of Rheumatology, National Centre for Rheumatic Diseases, Ratopul, Kathmandu, Nepal

**Egypt**

1. **Dr Hanan Mohamed Fathi**: Rheumatology and Autoimmune Diseases, Faculty of Medicine, Fayoum University, Faiyum, Egypt
2. **Dr Reem Hamdy A Mohammed**: Department of Rheumatology and Clinical Immunology, Kasr Alainy School of Medicine- Cairo University, Cairo, Egypt.

**Taiwan**

1. **Dr Yi-Ming Chen**: Division of Allergy, Immunology and Rheumatology, Department of Medical Research, Taichung Veterans General Hospital, Taiwan. blacklark@gmail.com

**United Arab Emirates**

1. **Dr Ghita Harifi**: Department of Rheumatology, Mediclinic Parkview Hospital, 3 Umm Suqeim St - Al Barsha Al Barsha South, Dubai, United Arab Emirates

**Saudi Arabia**

1. **Dr Lina El Kibbi**: Department of Rheumatology, Specialised Medical Center Hospital, Alfaisal University, Riyadh, Saudi Arabia
2. **Dr Hussein Mohammed Halabi**: Department of Medicine, King Faisal Specialist Hospital and Research Center, Jeddah, Saudi Arabia.

**Thailand**

- - - 1. **Dr P Akawatcharangura**: Department of Medicine, Queen Savang Vadhana Memorial Hospital, Chonburi, Thailand

1. **Dr. Wanruchada Katchamart:** 1. Division of Rheumatology, Department of Medicine, Faculty of Medicine Siriraj Hospital, Mahidol University, Thailand 2. Wanglang Road, Bangkok Noi, Bangkok, 10700, Thailand

**Venezuela**

1. **Dr. Yurilís Fuentes-Silva**: 1. Health Sciences School, University of Oriente– Bolivar Nucleus, Ciudad Bolivar, Venezuela. 2. Centro Clínico Universitario de Oriente, Ciudad Bolivar, Venezuela

**Paraguay**

1. **Dr. Karoll Cabriza:** Kinesiologa, Hospital de clinicas asuncion, Paraguay
2. **Dr.** **Jonathan Losanto**: Hospital de Clínicas, San Lorenzo, Paraguay
3. **Dr. Nelly Colaman**: Hospital de Clínicas, San Lorenzo, Paraguay

**Panama**

1. **Dr.** **Antonio Cachafeiro-Vilar**: Pacífica Salud-Hospital Punta Pacífica, Ciudad de Panamá, Panamá
2. **Dr. Generoso Guerra Bautista**: Centro de Investigación Marbella, Paitilla Panamá, Panamá
3. **Dr. Enrique Julio Giraldo Ho**: Rheumatology, Universidad de Panamá, República de Panamá, Panamá
4. **Dr. Raúl Agustín González**: Caja de Seguro Social de Panamá, Panamá

**Chile**

1. **Dr. Lilith Stange Nunez**: Rheumatology, University of Valparaiso, Pontifical Catholic University of Chile, Chile
2. **Dr. Cristian Vergara M**: Departamento de Medicina Interna, Escuela de Medicina Dirección de PostGrado y Post Título, Facultad de Medicina, Universidad de Valparaíso, Santiago, Chile

**Dominican Republic**

1. **Dr. Jossiell Then Báez**: Hospital Metropolitano de Santiago (HOMS), Santiago, Dominican Republic

**Honduras**

1. **Dr. Hugo Alonzo**: Jefe del Departamento de Medicina Interna en Hospital de Especialidades del Seguro Social en Tegucigalpa, Honduras
2. **Dr. Carlos Benito Santiago Pastelin**: Médico especialista, Instituto Hondureño del Seguro Social, Honduras.

**Argentina**

1. **Dr. Rodrigo García Salinas**: Rheumatology Unit, La Plata Italian Hospital, Buenos Aires, Argentina

**Guatemala**

1. **Dr. Alejandro Quiñónez Obiols**: Universidad Mariano Gálvez de Guatemala, Guatemala City, Guatemala
2. **Dr. Nilmo Chávez**: Instituto Guatemalteco de Seguridad Social - Universidad San Carlos de Guatemala, Ciudad de Guatemala, Guatemala
3. **Dr. Andrea Bran Ordóñez**: Hospital Universitario Esperanza, Guatemala City, Guatemala
4. **Dr. Sandra Argueta**: Rheumatology and Immunology, Universidad de San Carlos de Guatemala, Ciudad de Guatemala, Guatemala
5. **Dr. Daniel Quijivix**: Medicina Interna, Hidro Clinic, Quezaltenangro, Guatemala

**Cuba**

1. **Dr. Gil Alberto Reyes Llerena**: Surgical Medical Research Center (CIMEQ), Rheumatology Service, 216th Street and 11B, Siboney. Beach. Havana, Cuba

**Puerto Rico**

1. **Dr. Radames Sierra-Zorita**: University of Puerto Rico, School of Medicine, San Juan, Puerto Rico

**Costa Rica**

1. **Dr. Dina Arrieta**: Hospital México, Caja Costarricense del Seguro Social, San José de Costa Rica, Costa Rica
2. **Dr. Eduardo Romero Hidalgo**: Jackson's Memorial Medical Center, San Ramon, Alajuela, Costa Rica
3. **Dr. Ricardo Saenz**: Jefe Servicio Reumatología Hospital Dr. Calderón Guardia C.C.S.S, Costa Rica

**Nicaragua**

1. **Dr. Idania Escalante M**.: 1. Internal Medicine, Oscar Danilo Rosales School Hospital, Leon-Nicaragua. 2. Internal Medicine, National Autonomous University of Nicaragua, Managua, Nicaragua
2. **Dr.** **Roberto Morales**

**Equador**

1. **Dr. Wendy Calapaqui**: Instituto Ecuatoriano de Seguridad Social, Centro de atención ambulatoria “El Batán”, Quito, Ecuador
2. **Dr. Ivonne Quezada**: Hospital de Especialidades Eugenio Espejo, Quito, Ecuador

**Bolivia**

1. **Dr. Gabriela Arredondo**: Reumatóloga, Caja, Bancaria Estatal de Salud, Le Paz, Bolivia
