# Supplementary material for: COVAD survey 2 long-term outcomes: unmet need and protocol
Source: Rheumatol Int. 2022 Aug 14;42(12):2151–8. doi: 10.1007/s00296-022-05157-6 (PMC9376047; doi:10.1007/s00296-022-05157-6)
Supplement: Supplementary file 2 — Supplementary file2 (PDF 897 KB) [file 296_2022_5157_MOESM2_ESM.pdf]

COVID-19 Vaccination in Autoimmune Disease (CoVAD)-2 Study

Many countries are actively vaccinating their population against COVID-19 infection after large clinical trials showed the effectiveness and safety of the vaccine. However, the data on long-term effects and safety of the vaccine in patients with autoimmune disease is lacking. We are a group of doctors and scientists who want to study the effects of the COVID-19 vaccine in patients with autoimmune diseases in comparison to healthy individuals. Therefore, we request both individuals with autoimmune disease as well as those without any diagnosed autoimmune disease to take this survey. We invite eligible respondents to take this short survey irrespective of their vaccination status.

This survey takes less than seven minutes. Your responses are kept confidential and anonymous. We will analyze the data for publication in a medical journal, to discuss the long-term effects and safety of the COVID-19 vaccine.

* Did you receive a COVID-19 vaccine?

- ◯ Yes
- ◯ No

COVID-19 Vaccination in Autoimmune Disease (CoVAD)-2 Study

Vaccine hesitancy

* Why did you not have the vaccine?

- [ ] My doctor has advised against it
- [ ] Not available to me so far but I plan to have the vaccine as soon as possible
- [ ] I don’t believe in the science behind COVID vaccines
- [ ] Will not have the vaccine due to long-term safety concern or fear
- [ ] Planning to wait for more time/data regarding safety before I have the vaccine
- [ ] I have scheduled my vaccine, but have not received yet
- [ ] Not recommended as I had COVID-19 infection recently
- [ ] Unsure
- [ ] Other (please specify)

COVID-19 Vaccination in Autoimmune Disease (CoVAD)-2 Study

COVID vaccination

* How many COVID-19 vaccines have you received?
Count **booster** as a dose; so if you have received **2 regular vaccine doses and 1 booster**, then the answer would be **3 doses**.

- ◯ 1 dose
- ◯ 2 doses
- ◯ 3 doses
- ◯ 4 doses

COVID vaccination

* On which date did you receive the **first** dose of the vaccine?
If you do not remember the exact date, please choose the first date of the month.

Date

Date

DD/MM/YYYY

* Which COVID-19 vaccine did you receive for your **first** dose?

- Pfizer-BioNTech
- Oxford/Astra Zeneca
- Johnson & Johnson (J&J)
- Moderna
- Novavax
- Covishield (Serum Institute of India)
- Covaxin (Bharat Biotech)
- Sputnik
- Sinopharm
- Sinovac-CoronaVac
- I am not sure which one I received
- Other (please specify)

* Did you experience any minor (mild, not requiring medical attention) symptoms any time after 7 days of any vaccination? – choose multiple if more than 1 symptom. **Do not respond if symptoms occurred within seven days of vaccination and lasted less than 7 days.**

Minor symptoms are the ones that resolve within a few days with or without medication.

- [ ] **None**- I did not develop any symptoms after 7 days of vaccination
- [ ] Injection site (arm) pain and soreness
- [ ] Pain in all muscles of the body
- [ ] Body ache
- [ ] Joint pains
- [ ] Fever
- [ ] Chills
- [ ] Cough
- [ ] Difficulty in breathing or Shortness of breath
- [ ] Nausea/vomiting
- [ ] Headache
- [ ] Rash
- [ ] Fatigue
- [ ] Diarrhoea
- [ ] Abdominal pain
- [ ] High pulse rate or palpitations
- [ ] Rise in blood pressure
- [ ] Fainting
- [ ] Dizziness
- [ ] Chest pain
- [ ] Swelling in the extremities
- [ ] Weakness and tingling in the feet and legs
- [ ] Pricking or pins and needles sensations in the hands and feet
- [ ] Visual disturbances (loss of vision, blurring of vision, etc.)
- [ ] Bleeding/bruising on the body
- [ ] Petechial rash
- [ ] Other (please specify)

* Did you experience any major (severe or needing medical attention) symptoms any time after vaccination? – choose multiple if more than 1 symptom.

- [ ] **None**
- [ ] Anaphylaxis (severe, life threatening allergic reaction with shock)
- [ ] Marked difficulty in breathing
- [ ] Tongue swelling or throat closure
- [ ] Severe diffuse body rash (hives)
- [ ] Required hospitalisation
- [ ] Other severe symptoms (please specify)

If you required hospitalisation, please state the reason.

* How long (days) did your minor and major symptoms you marked above last?
Please select **0/None** from the dropdown menu if you did not experience any symptoms after the COVID-19 vaccination?

	1st dose
Minor	
Major	

COVID Vaccination

* On which date did you receive the **first** dose of the vaccine?
If you do not remember the exact date, please choose the first date of the month.

Date

Date

DD/MM/YYYY

* On which date did you receive the **second** dose of the vaccine?
If you do not remember the exact date, please choose the first date of the month.

Date / Time

Date

DD/MM/YYYY

* Which COVID-19 vaccine did you receive?

	1st dose	2nd dose
Pfizer-BioNTech	☐	☐
Oxford/Astra Zeneca	☐	☐
Johnson & Johnson (J&J)	☐	☐
Moderna	☐	☐
Novavax	☐	☐
Covishield (Serum Institute of India)	☐	☐
Covaxin (Bharat Biotech)	☐	☐
Sputnik	☐	☐
Sinopharm	☐	☐
Sinovac-CoronaVac	☐	☐
I am not sure which one I received	☐	☐

Other (please specify)

____

* Did you experience any minor (mild, not requiring medical attention) symptoms any time after 7 days of any vaccination? – choose multiple if more than 1 symptom. **Do not respond if symptoms occurred within seven days of vaccination and lasted less than 7 days.**

Minor symptoms are the ones that resolve within a few days with or without medication.

	1st dose	2nd dose
**None**- I did not develop any symptoms after 7 days of vaccination	☐	☐
Injection site (arm) pain and soreness	☐	☐
Pain in all muscles of the body	☐	☐
Body ache	☐	☐
Joint pains	☐	☐
Fever	☐	☐
Chills	☐	☐
Cough	☐	☐

	1st dose	2nd dose
Difficulty in breathing or Shortness of breath	☐	☐
Nausea/vomiting	☐	☐
Headache	☐	☐
Rash	☐	☐
Fatigue	☐	☐
Diarrhoea	☐	☐
Abdominal pain	☐	☐
High pulse rate or palpitations	☐	☐
Rise in blood pressure	☐	☐
Fainting	☐	☐
Dizziness	☐	☐
Chest pain	☐	☐
Swelling in the extremities	☐	☐
Weakness and tingling in the feet and legs	☐	☐
Pricking or pins and needles sensations in the hands and feet	☐	☐
Visual disturbances (loss of vision, blurring of vision, etc.)	☐	☐
Bleeding/bruising on the body	☐	☐
Petechial rash	☐	☐

Other (please specify)

* Did you experience any major (severe or needing medical attention) symptoms any time after any dose of vaccination? – choose multiple if more than 1 symptom.

	1st dose	2nd dose
**None**	☐	☐
Anaphylaxis (severe, life threatening allergic reaction with shock)	☐	☐
Marked difficulty in breathing	☐	☐
Tongue swelling or throat closure	☐	☐
Severe diffuse body rash (hives)	☐	☐
Required hospitalisation	☐	☐

Other severe symptoms (please specify)

If you required hospitalisation, please state the reason.

* How long (days) did your minor and major symptoms you marked above last?
Please select **0/None** from the dropdown menu if you did not experience any symptoms after the COVID-19 vaccination?

	1st dose	2nd dose
Minor		
Major		

COVID-19 Vaccination in Autoimmune Disease (CoVAD)-2 Study

COVID vaccination

* On which date did you receive the **first** dose of the vaccine?
If you do not remember the exact date, please choose the first date of the month.

Date

Date
DD/MM/YYYY

* On which date did you receive the **second** dose of the vaccine?
If you do not remember the exact date, please choose the first date of the month.

Date / Time

Date
DD/MM/YYYY

* Which COVID-19 vaccine did you receive?

	1st dose	2nd dose	3rd dose
Pfizer-BioNTech	☐	☐	☐
Oxford/Astra Zeneca	☐	☐	☐
Johnson & Johnson (J&J)	☐	☐	☐
Moderna	☐	☐	☐
Novavax	☐	☐	☐
Covishield (Serum Institute of India)	☐	☐	☐
Covaxin (Bharat Biotech)	☐	☐	☐
Sputnik	☐	☐	☐
Sinopharm	☐	☐	☐
Sinovac-CoronaVac	☐	☐	☐
I am not sure which one I received	☐	☐	☐

Other (please specify)

* On which date did you receive the **third** dose of the vaccine?
If you do not remember the exact date, please choose the first date of the month.

Date / Time

Date

DD/MM/YYYY

* Did you experience any minor (mild, not requiring medical attention) symptoms any time after 7 days of any vaccination? – choose multiple if more than 1 symptom. **Do not respond if symptoms occurred within seven days of vaccination and lasted less than 7 days.**

Minor symptoms are the ones that resolve within a few days with or without medication.

	1st dose	2nd dose	3rd dose
**None**- I did not develop any symptoms after 7 days of vaccination	☐	☐	☐
Injection site (arm) pain and soreness	☐	☐	☐

	1st dose	2nd dose	3rd dose
Pain in all muscles of the body	☐	☐	☐
Body ache	☐	☐	☐
Joint pains	☐	☐	☐
Fever	☐	☐	☐
Chills	☐	☐	☐
Cough	☐	☐	☐
Difficulty in breathing or Shortness of breath	☐	☐	☐
Nausea/vomiting	☐	☐	☐
Headache	☐	☐	☐
Rash	☐	☐	☐
Fatigue	☐	☐	☐
Diarrhoea	☐	☐	☐
Abdominal pain	☐	☐	☐
High pulse rate or palpitations	☐	☐	☐
Rise in blood pressure	☐	☐	☐
Fainting	☐	☐	☐
Dizziness	☐	☐	☐
Chest pain	☐	☐	☐
Swelling in the extremities	☐	☐	☐
Weakness and tingling in the feet and legs	☐	☐	☐
Pricking or pins and needles sensations in the hands and feet	☐	☐	☐
Visual disturbances (loss of vision, blurring of vision, etc.)	☐	☐	☐
Bleeding/bruising on the body	☐	☐	☐
Petechial rash	☐	☐	☐

Other (please specify)

____

* Did you experience any major (severe or needing medical attention) symptoms any time after any dose of vaccination? – choose multiple if more than 1 symptom.

	1st dose	2nd dose	3rd dose
**None**	☐	☐	☐
Anaphylaxis (severe, life threatening allergic reaction with shock)	☐	☐	☐
Marked difficulty in breathing	☐	☐	☐
Tongue swelling or throat closure	☐	☐	☐
Severe diffuse body rash (hives)	☐	☐	☐
Required hospitalisation	☐	☐	☐

Other severe symptoms (please specify)

____

If you required hospitalisation, please state the reason.

____

* How long (days) did your minor and major symptoms you marked above last?
Please select **0/None** from the dropdown menu if you did not experience any symptoms after the COVID-19 vaccination?

	1st dose	2nd dose	3rd dose
Minor			
Major			

COVID-19 Vaccination in Autoimmune Disease (CoVAD)-2 Study

COVID vaccination

* On which date did you receive the **first** dose of the vaccine?
If you do not remember the exact date, please choose the first date of the month.

Date

Date

DD/MM/YYYY

* On which date did you receive the **second** dose of the vaccine?
If you do not remember the exact date, please choose the first date of the month.

Date / Time

Date

DD/MM/YYYY

* On which date did you receive the **third** dose of the vaccine?
If you do not remember the exact date, please choose the first date of the month.

Date / Time

Date

DD/MM/YYYY

* On which date did you receive the **fourth** dose of the vaccine?
If you do not remember the exact date, please choose the first date of the month.

Date / Time

Date

DD/MM/YYYY

* Which COVID-19 vaccine did you receive?

	1st dose	2nd dose	3rd dose	4th dose
Pfizer-BioNTech	☐	☐	☐	☐
Oxford/Astra Zeneca	☐	☐	☐	☐
Johnson & Johnson (J&J)	☐	☐	☐	☐
Moderna	☐	☐	☐	☐
Novavax	☐	☐	☐	☐
Covishield (Serum Institute of India)	☐	☐	☐	☐
Covaxin (Bharat Biotech)	☐	☐	☐	☐
Sputnik	☐	☐	☐	☐
Sinopharm	☐	☐	☐	☐
Sinovac-CoronaVac	☐	☐	☐	☐
I am not sure which one I received	☐	☐	☐	☐

Other (please specify)

____

* Did you experience any minor (mild, not requiring medical attention) symptoms any time after 7 days of any vaccination? – choose multiple if more than 1 symptom. **Do not respond if symptoms occurred within seven days of vaccination and lasted less than 7 days.**

Minor symptoms are the ones that resolve within a few days with or without medication.

	1st dose	2nd dose	3rd dose	4th dose
**None**- I did not develop any symptoms after 7 days of vaccination	☐	☐	☐	☐
Injection site (arm) pain and soreness	☐	☐	☐	☐
Pain in all muscles of the body	☐	☐	☐	☐
Body ache	☐	☐	☐	☐
Joint pains	☐	☐	☐	☐
Fever	☐	☐	☐	☐
Chills	☐	☐	☐	☐
Cough	☐	☐	☐	☐

	1st dose	2nd dose	3rd dose	4th dose
Difficulty in breathing or Shortness of breath	☐	☐	☐	☐
Nausea/vomiting	☐	☐	☐	☐
Headache	☐	☐	☐	☐
Rash	☐	☐	☐	☐
Fatigue	☐	☐	☐	☐
Diarrhoea	☐	☐	☐	☐
Abdominal pain	☐	☐	☐	☐
High pulse rate or palpitations	☐	☐	☐	☐
Rise in blood pressure	☐	☐	☐	☐
Fainting	☐	☐	☐	☐
Dizziness	☐	☐	☐	☐
Chest pain	☐	☐	☐	☐
Swelling in the extremities	☐	☐	☐	☐
Weakness and tingling in the feet and legs	☐	☐	☐	☐
Pricking or pins and needles sensations in the hands and feet	☐	☐	☐	☐
Visual disturbances (loss of vision, blurring of vision, etc.)	☐	☐	☐	☐
Bleeding/bruising on the body	☐	☐	☐	☐
Petechial rash	☐	☐	☐	☐

Other (please specify)

* Did you experience any major (severe or needing medical attention) symptoms any time after any dose of vaccination? – choose multiple if more than 1 symptom.

	1st dose	2nd dose	3rd dose	4th dose
**None**	☐	☐	☐	☐
Anaphylaxis (severe, life threatening allergic reaction with shock)	☐	☐	☐	☐
Marked difficulty in breathing	☐	☐	☐	☐
Tongue swelling or throat closure	☐	☐	☐	☐
Severe diffuse body rash (hives)	☐	☐	☐	☐
Required hospitalisation	☐	☐	☐	☐

Other severe symptoms (please specify)

If you required hospitalisation, please state the reason.

* How long (days) did your minor and major symptoms you marked above last?
Please select **0/None** from the dropdown menu if you did not experience any symptoms after the COVID-19 vaccination?

	1st dose	2nd dose	3rd dose	4th dose
Minor				
Major				

COVID-19 Vaccination in Autoimmune Disease (CoVAD)-2 Study

Disease characteristics

* Do you have any of the following autoimmune conditions?

- [ ] **No autoimmune disease**
- [ ] Ankylosing spondylitis
- [ ] Crohn’s disease or ulcerative colitis (inflammatory bowel disease/IBD)
- [ ] Eosinophilic fasciitis
- [ ] Hemolytic anemia / idiopathic thrombocytopenic purpura (ITP)
- [ ] Mixed Connective Tissue
- [ ] Morphea
- [ ] Multiple sclerosis
- [ ] Myasthenia gravis
- [ ] Myositis/Anti-synthetase syndrome
- [ ] Overlap myositis with lupus or Sjögren or systemic sclerosis or rheumatoid arthritis
- [ ] Pernicious anemia
- [ ] Polymyalgia rheumatica (PMR)
- [ ] Psoriatic arthritis
- [ ] Rheumatoid arthritis
- [ ] Sjogren’s syndrome
- [ ] Systemic lupus erythematosus (lupus)
- [ ] Systemic sclerosis
- [ ] Thyroid (Hypothyroid or hyperthyroid)
- [ ] Type 1 Diabetes
- [ ] Undifferentiated Connective Tissue Disease
- [ ] Vasculitis
- [ ] Other autoimmune disease (please specify)

COVID-19 Vaccination in Autoimmune Disease (CoVAD)-2 Study

* What is your type of myositis?

- ◯ **I do not have myositis**
- ◯ Dermatomyositis
- ◯ Polymyositis
- ◯ Inclusion Body Myositis
- ◯ Anti-synthetase syndrome
- ◯ Necrotizing myositis
- ◯ Juvenile dermatomyositis

* Systemic sclerosis/Scleroderma

- ◯ **I do not have systemic sclerosis**
- ◯ My scleroderma INVOLVES skin thickening/tightening of my upper arms, thighs, chest and/or abdomen/belly
- ◯ My Scleroderma DOES NOT INVOLVE skin thickening/tightening of my upper arms, thighs, chest and/or abdomen/belly

* What is your type of vasculitis?

- ◯ **I do not have vasculitis**
- ◯ Giant cell arteritis
- ◯ Takayasu arteritis
- ◯ Polyarteritis nodosa
- ◯ ANCA-associated Vasculitis

COVID-19 Vaccination in Autoimmune Disease (CoVAD)-2 Study

* What is your type of ANCA associated vasculitis?

- ◯ Granulomatosis with Polyangiitis
- ◯ Microscopic polyangiitis
- ◯ Eosinophilic granulomatosis with polyangiitis

Disease characteristics

* Who confirmed your autoimmune disease diagnosis?

- [ ] Rheumatologist
- [ ] Neurologist
- [ ] Dermatologist
- [ ] Hematologist
- [ ] Physician/Medicine doctor (internal medicine, Internist)
- [ ] Primary care physician (PC), or family doctor or general practitioner (GP).
- [ ] Other doctor (please specify)

* In which year were you diagnosed with autoimmune disease?

* Did you experience any change in the status of your autoimmune disease after the **second** dose of the COVID-19 vaccine?

- ( ) No
- ( ) Yes, my autoimmune disease related symptoms worsened
- ( ) Yes, my autoimmune disease related symptoms improved.
- ( ) I did not take the 2nd dose/any dose of the vaccine

COVID-19 Vaccination in Autoimmune Disease (CoVAD)-2 Study

Disease flare after vaccination

* When did you experience worsening of your autoimmune disease?
If you do not remember the exact date, please choose the first date of the month.

Date / Time

Date

DD/MM/YYYY

* Mark only significant symptoms you experienced due to worsening that changed your day-to-day life functioning.

- [ ] Rashes
- [ ] Muscle weakness
- [ ] Muscle pain
- [ ] Joint pain or swelling in hands
- [ ] Pain in shoulders and hips
- [ ] Joint pain or swelling of other joints
- [ ] Raynaud's (blue, or white discolouration with or without pain in the fingers on exposure to cold)
- [ ] Skin thickening of hands
- [ ] Skin thickening in new areas of the body not previously affected
- [ ] Fingertip ulcers or pits
- [ ] Shortness of breath
- [ ] Chest pain
- [ ] Difficulty in swallowing
- [ ] Fever
- [ ] Fatigue
- [ ] Dry eyes
- [ ] Dry mouth
- [ ] Oral or nasal ulcers (sores)
- [ ] Severe loss of hair or in bald spots
- [ ] Headache due to disease
- [ ] Active kidney disease from autoimmune disease
- [ ] Elevated muscle enzyme in blood (high CK or creatine kinase level)
- [ ] Elevated inflammatory markers in blood (high ESR or CRP)
- [ ] Other (please specify)

Rashes and arthritis after vaccination

* What kind of rashes did you notice?

- [ ] I did not have rashes
- [ ] Red rash around the eyes
- [ ] Red rashes on the knuckles
- [ ] Rashes on the knees
- [ ] Rashes on the things or/and hips
- [ ] Red rash on the cheeks
- [ ] Red rash in the V area of the chest
- [ ] Red rashes on the outside of arms and/or forearm
- [ ] Mechanic's hand (rough thick scaly skin on the fingers)
- [ ] Other
- [ ] Other red rashes (please specify)

* If you have swelling in your joints, how many joints are swollen? (Do not count joints with bony enlargement/swelling)

- ◯ I did not have swelling in joints
- ◯ 1-2 swollen joints
- ◯ 3-5 swollen joints
- ◯ 5 or more swollen joints

* Did you modify medicines taken for your underlying autoimmune disease for these worsening symptoms?

- [ ] No
- [ ] Switched to new medicine for my autoimmune disease
- [ ] Added new medicine for my autoimmune disease
- [ ] Started or increased the dose of steroids (prednisone, prednisolone, medrol, wysolone, omnacortil)

COVID-19 Vaccination in Autoimmune Disease (CoVAD)-2 Study

Medicines record

* Were you on any of the following immunosuppressive medications before COVID-19 vaccination?

- [ ] None
- [ ] Methotrexate
- [ ] Mycophenolate mofetil or mycophenolic acid
- [ ] Azathioprine
- [ ] Hydroxychloroquine
- [ ] Sulfasalazine
- [ ] Leflunomide
- [ ] Oral Tacrolimus
- [ ] Cyclosporine
- [ ] IV immunoglobulin (IVIg) or subcutaneous immunoglobulin (SQIg)
- [ ] Cyclophosphamide (Cytoxan)
- [ ] Rituximab
- [ ] Anti TNF agents (infliximab, adalimumab, certolizumab, golimumab, etanercept)
- [ ] Other Biologics (specify)
- [ ] JAK inhibitors (Tofacitinib, Baricitinib, Upadacitinib)
- [ ] Other immunosuppressant/immunomodulator medicines (Specify)
- [ ] Other biologics (please specify)

____

* Were you on any steroids (prednisone, Medrol, prednisolone, wysolone, omacortil, etc) before COVID-19 vaccination?

- ( ) No steroids
- ( ) Yes, < 10 mg a day
- ( ) Yes, 10 mg - 20 mg a day
- ( ) Yes, > 20 mg a day

* Did you require an increase in the dose of any of these immunosuppressant medications, or starting a new immunosuppressant medicine any time after receiving the **second** dose of the COVID-19 vaccine?
Do **NOT** include if the dose was decreased or the medication stopped.

- [ ] Not applicable (Did not take the second dose)
- [ ] No
- [ ] Steroids
- [ ] Methotrexate
- [ ] Mycophenolate mofetil or mycophenolic acid
- [ ] Azathioprine
- [ ] Hydroxychloroquine
- [ ] Sulfasalazine
- [ ] Leflunomide
- [ ] Oral Tacrolimus
- [ ] Cyclosporine
- [ ] IV immunoglobulin (IVIg) or subcutaneous immunoglobulin (SQIg)
- [ ] Cyclophosphamide (Cytoxan)
- [ ] Rituximab
- [ ] Anti TNF agents (infliximab, adalimumab, certolizumab, golimumab, etanercept)
- [ ] Other Biologics (specify)
- [ ] JAK inhibitors (Tofacitinib, Baricitinib, Upadacitinib)
- [ ] Other immunosuppressant/immunomodulator medicines
- [ ] Other biologics (please specify)

* How has your disease been before the first dose and after the second dose of the COVID-19 vaccine?

	Before 1st dose	After 2nd dose
I did not take the second dose	☐	☐
My disease is inactive or in remission	☐	☐
My disease is active but stable and manageable	☐	☐
My disease is active and improving	☐	☐
My disease is active and worsening	☐	☐
I am not sure	☐	☐

Other (please specify)

* Did you develop symptoms of and get diagnosed with a new autoimmune disease after receiving any dose of the COVID-19 vaccine?

- ◯ Yes
- ◯ No

COVID-19 Vaccination in Autoimmune Disease (CoVAD)-2 Study

* Would you like us to get in touch with you with future surveys to understand more about your new autoimmune disease after COVID-19 vaccination?

- Yes
- No

Co-morbid conditions

* Has a doctor/physician ever diagnosed you with any of the following conditions?

- [ ] **None**
- [ ] Coronary Heart Disease/Ischemic Heart Disease
- [ ] Diabetes Mellitus (Type 2)
- [ ] Hypertension
- [ ] Stroke
- [ ] Chronic Kidney Disease
- [ ] Chronic Liver Disease
- [ ] Chronic Obstructive Pulmonary Disease (COPD) (Emphysema, Bronchitis, etc.)
- [ ] Epilepsy
- [ ] HIV-AIDS
- [ ] Bronchial Asthma
- [ ] Other (please specify)

____

* Has a doctor/physician ever diagnosed you with any of the following conditions?

- [ ] **None**
- [ ] Anxiety
- [ ] Bipolar disorder
- [ ] Depression
- [ ] Eating disorders
- [ ] Insomnia
- [ ] Schizophrenia
- [ ] Substance use disorders
- [ ] Other (please specify)

____

COVID-19 Vaccination in Autoimmune Disease (CoVAD)-2 Study

COVID-19 infection

* Did you ever test positive for COVID-19?

- Yes, once
- Yes, twice
- Yes, three times or more
- No

COVID-19 infection

* When did you test positive for COVID-19?
If you do not remember the exact date, please choose the first date of the month.

Date

Date

DD/MM/YYYY

* What were your symptoms due to COVID-19 infection?

- [ ] **None** (asymptomatic– no symptoms)
- [ ] Fever
- [ ] Fatigue
- [ ] Muscle aches
- [ ] Joint pains
- [ ] Cough
- [ ] Difficulty in breathing or Shortness of breath
- [ ] Loss of smell
- [ ] Loss of taste
- [ ] Running nose
- [ ] Congestion
- [ ] Throat pain/scratchiness
- [ ] Chest pain
- [ ] Diarrhoea
- [ ] Headache
- [ ] Oral ulcers
- [ ] Nausea/vomiting
- [ ] Abdominal/Belly pain
- [ ] Skin rashes
- [ ] Other (please specify)

* Did you require hospitalization, ICU admission or oxygen supplementation due to COVID-19 infection? (**Hospitalization is not the same as being kept in an isolation centre/ facility**)

- [ ] Hospitalization
- [ ] Intensive Care Unit (ICU) or Other High Dependence Unit
- [ ] Oxygen supplementation
- [ ] **No**, I did not require any of these

* Did you receive any advanced treatment for COVID-19?

- [ ] **No**
- [ ] Monoclonal antibody- Casirivimab
- [ ] Monoclonal antibody- Imdevimab (Ronapreve)
- [ ] Molnupiravir (Merck's oral pill for COVID-19)
- [ ] Paxlovid (nirmatrelvir with ritonavir) (Pfizer's oral pill for COVID-19)
- [ ] Not sure
- [ ] Other (please specify)

* How long (days) did it take for the symptoms you reported above to completely resolve?

0 — 365

* Did your autoimmune disease flare-up after COVID-19 infection?

- ○ Yes
- ○ No
- ○ I am not sure
- ○ I do not have an autoimmune disease

COVID-19 infection

* When did you test positive for COVID-19 the **first** time?
If you do not remember the exact date, please choose the first date of the month.

Date

Date

DD/MM/YYYY

* When did you test positive for COVID-19 the **second** time?
If you do not remember the exact date, please choose the first date of the month.

Date

Date

DD/MM/YYYY

* What were your symptoms due to COVID-19 infection?

	1st infection	2nd infection
**None** (asymptomatic– no symptoms)	☐	☐
Fever	☐	☐
Fatigue	☐	☐
Muscle aches	☐	☐
Joint pains	☐	☐
Cough	☐	☐
Difficulty in breathing or Shortness of breath	☐	☐
Loss of smell	☐	☐
Loss of taste	☐	☐
Running nose	☐	☐
Congestion	☐	☐
Throat pain/scratchiness	☐	☐
Chest pain	☐	☐
Diarrhoea	☐	☐
Headache	☐	☐
Oral ulcers	☐	☐
Nausea/vomiting	☐	☐
Abdominal/Belly pain	☐	☐
Skin rashes	☐	☐

Other (please specify)

* Did you require hospitalization, ICU admission or oxygen supplementation due to COVID-19 infection? (**Hospitalization is not the same as being kept in an isolation centre/ facility**)

	1st infection	2nd infection
Hospitalization	☐	☐
Intensive Care Unit (ICU) or Other High Dependence Unit	☐	☐
Oxygen supplementation	☐	☐
**No**, I did not require any of these	☐	☐

Other (please specify)

[ ]

* Did you receive any advanced treatment for COVID-19?

	1st infection	2nd infection
**No**	☐	☐
Monoclonal antibody-Casirivimab	☐	☐
Monoclonal antibody-Imdevimab (Ronapreve)	☐	☐
Molnupiravir (Merck's oral pill for COVID-19)	☐	☐
Paxlovid (nirmatrelvir with ritonavir) (Pfizer's oral pill for COVID-19)	☐	☐
Not sure	☐	☐

Other (please specify)

[ ]

* How long (days) did it take for the symptoms you reported above to completely resolve after the **first** COVID-19 infection?

0 365

* How long (days) did it take for the symptoms you reported above to completely resolve after the **second** COVID-19 infection?

0 365

* Did your autoimmune disease flare-up after COVID-19 infection?

	1st infection	2nd infection
Yes	☐	☐
No	☐	☐
I am not sure	☐	☐

Antibodies protecting from COVID infection

* Were you ever tested for COVID-19 autoantibodies?

- ◯ Yes
- ◯ No

COVID-19 Vaccination in Autoimmune Disease (CoVAD)-2 Study

Antibodies protecting from COVID infection

* When were you tested for COVID-19 autoantibodies?
If you do not remember the exact date, please choose the first date of the month. (Most **recent** test date if taken **multiple** times)

Date

Date

DD/MM/YYYY

* What was the result of the COVID-19 autoantibody test? (Most **recent** test results if taken **multiple** times)

- ◯ Antibodies were present
- ◯ Antibodies were absent
- ◯ I am not sure

COVID-19 Vaccination in Autoimmune Disease (CoVAD)-2 Study

Current health status and quality of life

* What is your current health status?
Please respond to each item by marking **one circle per row**.

	Excellent	Very good	Good	Fair	Poor
In general, would you say your health is:	○	○	○	○	○
In general, would you say your quality of life is:	○	○	○	○	○
In general, how would you rate your physical health?	○	○	○	○	○
In general, how would you rate your mental health, including your mood and your ability to think?	○	○	○	○	○
In general, how would you rate your satisfaction with your social activities and relationships?	○	○	○	○	○
In general, please rate how well you carry out your usual social activities and roles. (This includes activities at home, at work and in your community, and responsibilities as a parent, child, spouse, employee, friend, etc.)	○	○	○	○	○

* To what extent are you able to carry out your everyday physical activities such as walking, climbing stairs, carrying groceries, or moving a chair?

Completely	Mostly	Moderately	A Little	Not At All
○	○	○	○	○

* What is your current status of physical health?
Please respond to each question or statement by marking **one circle per row**.

	Not at all	Very little	Somewhat	Quite a lot	Cannot do
Does your health now limit you in doing vigorous activities, such as running, lifting heavy objects, participating in strenuous sports?	○	○	○	○	○
Does your health now limit you in walking more than a mile (1.6 km)?	○	○	○	○	○
Does your health now limit you in climbing one flight of stairs?	○	○	○	○	○
Does your health now limit you in lifting or carrying groceries?	○	○	○	○	○
Does your health now limit you in bending, kneeling, or stooping?	○	○	○	○	○

* What is your current status of physical health?
Please respond to each question or statement by marking **one circle per row**.

	Without any difficulty	With a little difficulty	With some difficulty	With much difficulty	Unable to do
Are you able to do chores such as vacuuming or yard work?	○	○	○	○	○
Are you able to dress yourself, including tying shoelaces and buttoning your clothes?	○	○	○	○	○
Are you able to shampoo your hair?	○	○	○	○	○
Are you able to wash and dry your body?	○	○	○	○	○
Are you able to sit on and get up from the toilet?	○	○	○	○	○

* In the past seven days, how often have you been bothered by emotional problems such as feeling anxious, depressed, or irritable?

Never	Rarely	Sometimes	Often	Always
○	○	○	○	○

* In the past seven days, how would you rate your fatigue on average?

None	Mild	Moderate	Severe	Very Severe
○	○	○	○	○

* During the past 7 days
(Please respond to each question or statement by marking **one circle per row**.)

	Not at all	A little bit	Somewhat	Quite a bit	Very much
I feel fatigued	○	○	○	○	○
I have trouble starting things because I am tired	○	○	○	○	○

* In the past 7 days
(Please respond to each question or statement by marking **one circle per row**.)

	Not at all	A little bit	Somewhat	Quite a bit	Very much
How run-down did you feel on average?	○	○	○	○	○
How fatigued were you on average?	○	○	○	○	○

* In the past seven days, how would you rate your pain on average?

0 (No pain)	1	2	3	4	5	6	7	8	9	10 (Worst Imaginable Pain)
○	○	○	○	○	○	○	○	○	○	○

* Which part of your body would you describe as painful?

- [ ] Large joints (eg., hip, knee, shoulder, elbow, etc.)
- [ ] Small joints (eg., finger joints, etc.)
- [ ] Back muscles
- [ ] Arm muscles (eg., biceps, triceps, etc.)
- [ ] Leg muscles (eg., quadriceps, hamstrings, etc.)
- [ ] Finger muscles
- [ ] Everywhere
- [ ] Other (please specify)

COVID-19 Vaccination in Autoimmune Disease (CoVAD)-2 Study

Just a few final questions about you!

* Ethnicity?

- Caucasian (White)
- African American or of African origin (Black)
- Asian
- Hispanic
- Native American/Indigenous/Pacific Islander
- Do not wish to disclose
- Other (please specify)

* Which country do you live in?

* Gender

- Male
- Female
- Do not wish to disclose
- Other (please specify)

COVID-19 Vaccination in Autoimmune Disease (CoVAD)-2 Study

* Are you pregnant or lactating/breastfeeding currently?

- ◯ Yes, I am pregnant
- ◯ Yes, I am lactating/breastfeeding

* What is your estimated date of delivery/date of child birth? (whichever is applicable)
If you do not remember the exact date, please choose the first date of the month.

Date

Date

DD/MM/YYYY

* Age?

18 100
